# Cooperative Repair for Laser-Induced Graphene via Modified Poly-phenylamine and Fe^2+^ for Thermal-Conductive Gels

**DOI:** 10.3390/gels12090835

**Published:** 2026-09-11

**Authors:** Nan Jiang, Guomin Ding, Bowen Yang, Shuai Liu, Luyao Wang, Zihan Li, Xu Han, Qilin Mei

**Affiliations:** School of Materials Science and Engineering, Wuhan University of Technology, No.122, Luoyu Road, Wuhan 430070, China; jignanw@163.com (N.J.);

**Keywords:** laser-induced graphene, poly-phenylamine, composite precursor, cooperative effect, thermal-conductive gel

## Abstract

Laser-induced graphene (LIG) has great potential for multiple applications because of its large specific surface area, facile fabrication process, and tunable properties. However, abundant lattice defects severely degrade its conductivity. Herein, from an innovative perspective of precursor design, the poly-phenylamines (P-PAs) with improved solubility and strong light absorption were synthesized, which act as an intercalated polymer for graphene oxide (GO) nanosheets. On this basis, the composite precursors show remarkably enhanced photothermal conversion capability and a compact stacked structure. These bring a 60% reduction in I_D_/I_G_ in LIG after laser irradiation. To explain the above phenomenon, an isolation effect induced by the compact stacking precursor is proposed based on experimental results. Furthermore, the cooperative effect between P-PAs and Fe^2+^ is introduced, and a fluffy LIG aerogel with the lowest I_D_/I_G_ ratio of 0.17 is prepared, which is barely achievable in conventional LIGs. When the obtained graphene aerogel is compounded with PDMS, the as-prepared thermal-conductive composite gel reaches a thermal conductivity of 1.05 W·m^−1^·K^−1^ and an ultralow interfacial thermal resistance of 37.2 mm^2^·K·W^−1^ under a low graphene loading of 3.3 wt%. This intercalation strategy in GO precursor supplies a new route for preparing high-quality LIGs and thermal-conductive gels, which show great application prospects in thermal management devices.

## 1. Introduction

Owing to its tunable properties, large specific surface area, and efficient fabrication process, laser-induced graphene (LIG) has emerged as a revolutionary approach for preparing porous graphene aerogels and their corresponding thermally conductive gels [1,2,3,4,5]. In contrast to common precursors such as polyimide (PI) [6,7,8], phenolic resin and biomass materials [9,10,11], graphene oxide (GO) undergoes more facile transformation under laser irradiation because it possesses an analogous molecular structure to graphene [12,13]. However, LIG derived from GO still suffers from abundant lattice defects, so it demonstrates an inferior thermal conductivity. This is mainly attributed to the existing fabrication process and the natural property of GO. Specifically, most laser-induced conversion processes are performed under atmospheric conditions, which fail to restrain oxidation of LIG during laser processing. Due to the weak light absorbance of GO nanosheets, their poor photothermal conversion yields a low laser-induced temperature, which further accounts for the insufficient lattice repair of the as-obtained graphene. Although a higher-intensity laser is usually employed, the GO will be seriously etched rather than repaired.

To improve the quality of LIG, special gas environments are generally employed during laser irradiation. Research reveals that Argon or Argon/H_2_ atmospheres exhibit obvious effectiveness in assisting restoration of lattice defects of graphene [9,14]. However, these modification strategies inevitably increase fabrication complexity and production costs, while restricting the dimensions of as-prepared LIG. Besides, metals or metal ions can be utilized as catalysts to synthesize well-crystallized LIG, as experimentally demonstrated in previous studies [15,16,17,18,19]. But excessive metal will cause the precipitation of GO, which severely deteriorates the stacked state and mechanical properties of the GO precursor. In summary, the existing methods for enhancing the quality of LIG are predominantly focused on adjusting external conditions, including laser parameters [20,21], processing environment, or catalyst assistance [9,22]. However, the effect of precursor microstructure on the lattice restoration of LIG is often overlooked in previous studies. Recently, the modification of the material system for LIG has gained increasing popularity among researchers due to its simplicity and methodological diversity [20]. Especially the, appropriate polymers and their intercalation design are expected to effectively improve the quality of LIG. Unfortunately, the correlation between precursor characteristics and final LIG quality remains poorly explored, and targeted structural design for obtaining a more ordered structure has not been reported to date.

Herein, to achieve homogeneous intercalation with GO, the low-molecular-weight, modified poly-phenylamines (P-PAs) with improved solubility and strong light absorption were designed and synthesized in this study. By means of the as-prepared P-PAs, the GO composite precursors with tight interlayer bonding were fabricated through evaporation-induced co-assembly, and they showed a remarkable enhancement of the photothermal effect. Based on this, the quality of the obtained LIG was improved without external assistance, and the I_D_/I_G_ level of graphene was reduced by 60%. The mechanism underlying lattice repair of graphene facilitated by this composite precursor was specifically explored. Furthermore, Fe^2+^ was introduced as a catalyst. Benefiting from the cooperative effect between P-PAs and Fe^2+^, a fluffy LIG aerogel was finally obtained, with a decreased I_D_/I_G_ ratio of 0.17. Such a low defect level could hardly be achieved through conventional LIG-fabrication methods. By means of this aerogel, the thermal-conductive gel (TG) composited with PDMS achieved a high thermal conductivity of 1.05 W·m^−1^·K^−1^ and an ultralow interfacial thermal resistance of 37.2 mm^2^·K·W^−1^ at the graphene content of only 3.3 wt%. Therefore, the proposed fabrication strategy provides a new way for repairing the defects in LIG, and the resulting TG can serve as a promising candidate for a thermal-transport system.

## 2. Results and Discussion

### 2.1. Preparation of Modified P-PAs

**Figure 1:**
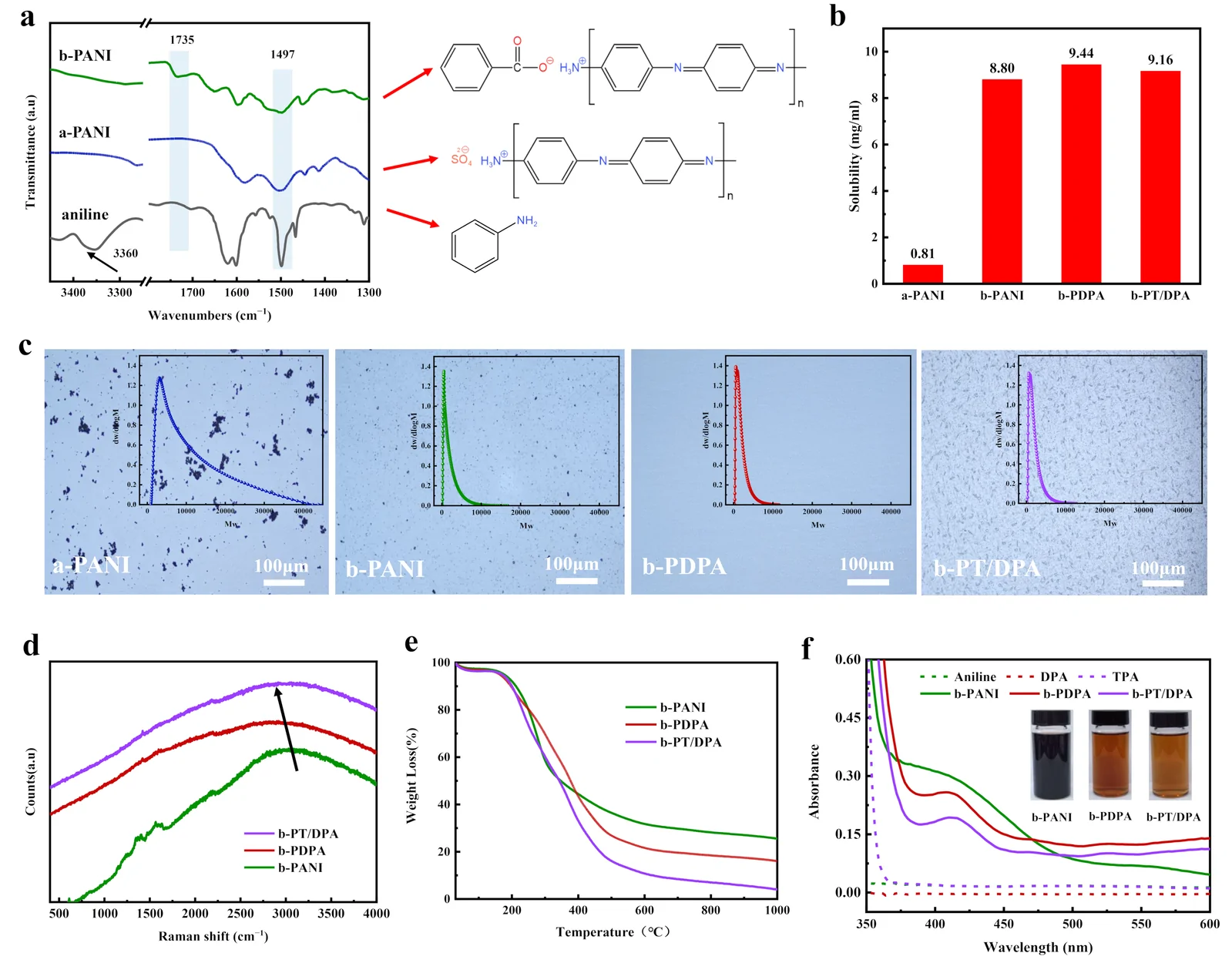
(**a**) The FTIR curves of different PANIs and aniline. (**b**) The solubility of different P-PAs. (**c**) Microscopic comparison of dried polymers and their molecular weight distribution. (**d**) The Raman curves of different P-PAs; (**e**) Thermogravimetric analysis curves of different P-PAs. (**f**) The UV-Vis spectra of different P-PAs and corresponding monomers.

According to our prior work, aromatic rings can provide a repairing effect on graphene under high-temperature conditions [23]. Meanwhile, it can achieve good compatibility with GO nanosheets via π-π interaction [24]. Therefore, P-PAs with lots of benzene structures were selected as intercalated polymers in this study. However, due to their intrinsic chemical characteristics, traditional P-PAs, such as polyaniline (PANI), exhibit poor solubility in the majority of solvents, including water and ethanol, which are common solvents for dispersing GO nanosheets [25,26]. To resolve the poor-solubility issue, the molecular structure of P-PAs was modified by adopting organic Benzoyl peroxide (BPO) instead of traditional ammonium peroxodisulfate (APS) as an initiator. The FTIR results show that, under the same conditions, both BPO and APS can initiate the polymerization of aniline monomers, so the N-H stretching vibration peaks in aniline monomers at 3360 cm^−1^ [27] disappeared in both APS-initiated PANI (a-PANI) and BPO-initiated PANI (b-PANI) owing to hydrogen proton deprotonation (Figure 1a). Besides, the C=C stretching vibration of the benzene ring at 1497 cm^−1^ in a-PANI and b-PANI becomes weaker compared with that in aniline monomers, which is mainly attributed to the partial transformation of benzene rings into quinone rings, demonstrating a highly oxidized state of the obtained PANIs [25,28]. Moreover, compared with that of a-PANI, a characteristic absorption peak at 1735 cm^−1^, assigned to the absorption vibration of C=O [29], can be found in the FTIR spectrum of b-PANI. We suppose that this is primarily attributed to the residues of the BPO initiators, which bind to the PANI chains based on the polymerization mechanism of PANI [30,31,32], as shown in Figure 1a.

In addition to molecular structure changes, the molecular weight of the above PANIs also shows significant differences. As shown in the inset of Figure 1c, the b-PANI shows an obviously lower weight-average molecular weight (M_W_) of 1078 than a-PANI (M_W_~6560). This is because BPO shows weaker oxidizing capacity compared with APS, which leads to inferior initiation activity [30,31,32]. Due to the aforementioned molecular differences, a-PANI and b-PANI display markedly different solubility in ethanol-water mixed solvent (volume ratio 9:1). As demonstrated in our previous work, this solvent system enables efficient evaporation-induced self-assembly of GO nanosheets [33]. The result shows that the solubility value of b-PANI reaches up to 8.80 mg mL^−1^, over ten-fold higher than that of a-PANI (0.81 mg mL^−1^, Figure 1b). This increased solubility enables broad tunability of the proportion between intercalated polymers and GO within composite precursors. The improved solubility of b-PANI originates from grafted organic residues of BPO, which increase intermolecular steric hindrance, and the lower molecular weight also facilitates dissolution. More importantly, the solubility exerts an influence on the agglomeration behavior of polymers following solvent evaporation. As shown in Figure 1c, after the solvent evaporated, a-PANI forms a few large aggregates. In contrast, b-PANI exists as many fine particles. Therefore, it is clear that the improved solubility will promote the formation of homogeneous composite GO precursors in later processes.

Based on the above method, other aniline-based monomers, including diphenylamine (DPA) and triphenylamine (TPA), were also polymerized using BPO as initiator. It should be noted that due to the serious steric hindrance, TPA cannot be homopolymerized on its own. Therefore, a copolymerization containing DPA and TPA (100:4 by weight) was adopted. The obtained polymers were named b-PDPA and b-PT/DPA, respectively. The FTIR results show that changes in characteristic peaks (N-H, C=C, and C=O) also occur (Figure S1) for b-PDPA and b-PT/DPA, indicating the successful synthesis of the corresponding polymers. Moreover, b-PDPA and b-PT/DPA are also low-molecular-weight polymers, which have smaller Mw of 1274 and 1302 (insets of Figure 1c), respectively, compared with corresponding APS initiating polymers (Figure S2). The solubility of b-PDPA and b-PT/DPA in ethanol-water solvent reaches 9.44 mg mL^−1^ and 9.16 mg mL^−1^, respectively. In our opinion, the highest solubility of b-PDPA can be explained as follows. Although b-PDPA has a higher Mw than b-PANI, because of the large relative molecular mass of DPA monomers, its average degree of polymerization (DP, 5.18) is lower than that of b-PANI (DP ~6.46). Besides, the strong steric hindrance derived from large DPA molecules enlarges the spacing between b-PDPA chains, weakening intermolecular interactions, which can also improve solubility. The weaker intermolecular force in b-PDPA is verified by its lower shift than that in b-PANI in Raman spectra [34] (Figure 1d). From this perspective, the weakest intermolecular interactions can be deduced in b-PT/DPA from the lowest Raman shift. However, even though b-PT/DPA has the smallest DP of 5.09, it still shows worse solubility than b-PDPA. This is due to the fact that TPA monomers possess poorer solubility than DPA monomers, resulting in a naturally low solubility for b-PT/DPA. Furthermore, the morphology of the dried P-PAs was investigated. As shown in Figure 1c, many smaller particles occur in dried b-PT/DPA than in dried b-PANI, and no aggregates are observed in dried b-PDPA.

Besides, the thermal stability of P-PAs was tested by thermogravimetric analysis. As shown in Figure 1e, the thermal decomposition temperatures of b-PANI, b-PDPA, and b-PT/DPA are 195.5 °C, 192.8 °C, and 190.1 °C, respectively. The final residue rate of b-PANI, b-PDPA, and b-PT/DPA is 25.54%, 16.04%, and 4.06%, respectively. The above differences in thermal stability are consistent with the DP and intermolecular forces of P-PAs. Additionally, benefiting from the conjugated interaction among benzene or quinone structures, the P-PAs display markedly deeper color compared to the corresponding monomers, which barely absorb light. The color of polymer solutions decreases progressively from b-PANI, b-PDPA to b-PT/DPA (inset in Figure 1f). Their absorbances were determined via UV–vis spectra, and the results showed that the absorbance intensity was consistent with the visual color, and b-PANI exhibited the strongest absorbance in the range of 370–470 nm (Figure 1f).

### 2.2. Preparation and Characterization of P-PA/GO Precursors

**Figure 2:**
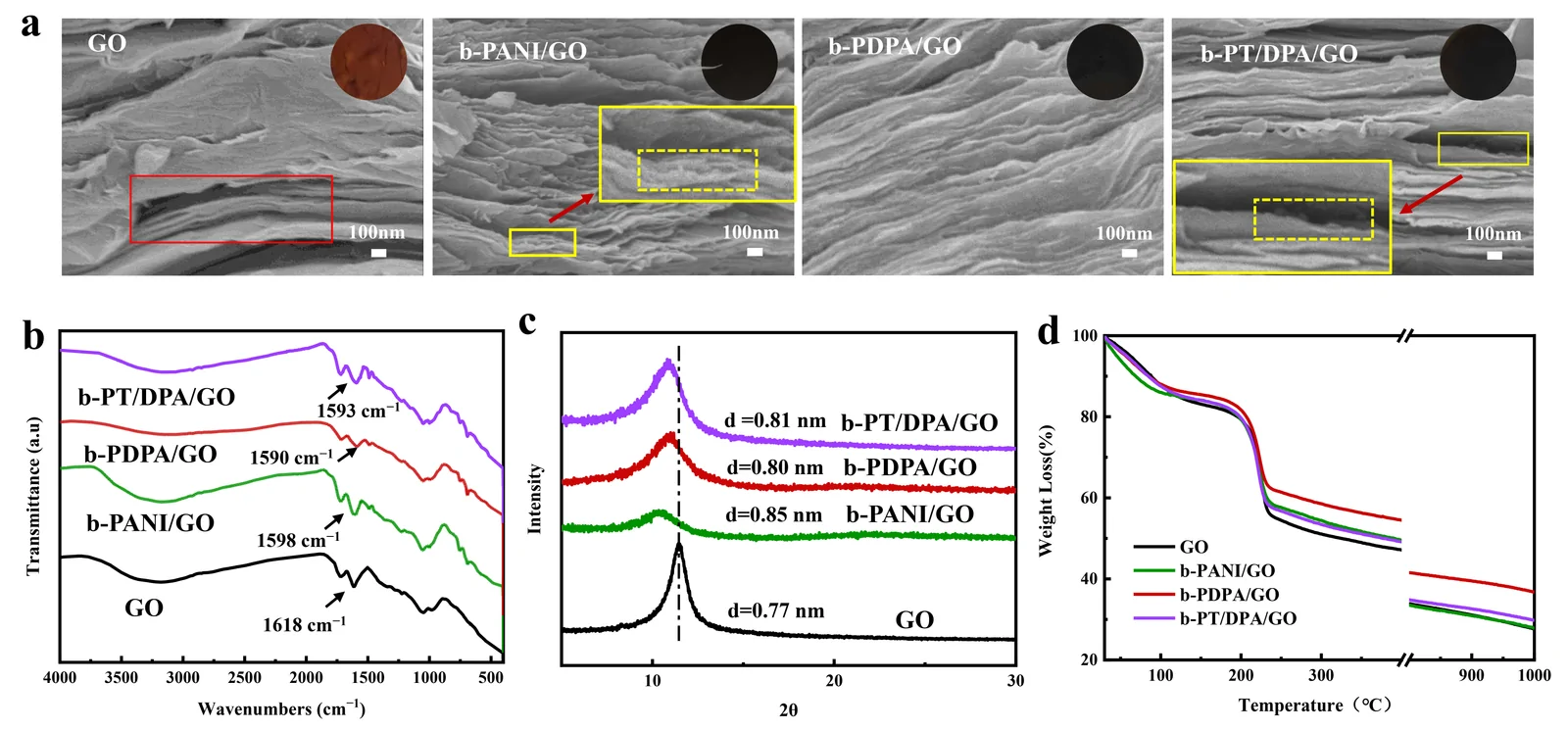
(**a**) The cross-sectional SEM of different composite precursors. (**b**) The FTIR curves of different composite precursors. (**c**) The XRD curves of different composite precursors. (**d**) The thermogravimetric analysis curves of different composite precursors.

By means of the improved solubility, the as-synthesized P-PAs were mixed with GO nanosheets in an ethanol-water solvent, and then self-assembled via an evaporation-induced process to fabricate corresponding composite precursors with a P-PAs-to-GO mass ratio of 1:4 based on our reported techniques [33]. The obtained precursors were named as b-PANI/GO, b-PDPA/GO, and b-PT/DPA/GO, respectively. The composite state between the polymer and GO was characterized by observing their cross-section using a scanning electron microscope (SEM). The results show that the fracture surface of pure GO precursors exhibits severe irregular morphology, and evident delamination can be observed between GO nanosheets (red box), indicating weak interlayer bonding (Figure 2a). In contrast, upon the incorporation of P-PAs, the cross-section of the composite precursors becomes smooth. We believe this is primarily because P-PAs improve the binding force between nanosheets due to their strong π-π interactions with GO, which is consistent with the original intention in selecting P-PA as an intercalant. The increased interaction can be confirmed by changes in the FTIR spectrum. As shown in Figure 2b, the peak at 1618 cm^−1^ in the spectrum of GO is assigned to the C=C stretching vibration of aromatic rings [35,36]. It redshifts to 1598 cm^−1^, 1590 cm^−1^, and 1593 cm^−1^ for b-PANI/GO, b-PDPA/GO, and b-PT/DPA/GO, respectively, which is because the strong π-π interactions between P-PA and GO extend the π-electron delocalized region and dilute the electron density in C=C bonds, so that the bond-stretching force constant is reduced [37].

Besides, the different P-PAs show different impacts on the layered structure in composite precursors. As shown in Figure 2a, b-PANI and b-PT/DPA form some agglomerates between the GO layers (yellow boxes), whereas b-PDPA presents a continuous distribution in the composite precursor, resulting in a more compact stack structure. This is consistent with the dried states of the different P-PAs. As a comparison, the traditional P-PAs initiated by APS present more serious agglomeration after compositing with GO (Figure S3). The above stacked states also affect the interlayer spacing between the GO nanosheets. The XRD pattern shows that the interlayer spacing of the pure GO precursor is 0.77 nm. With the introduction of polymers, the interlayer spacing of b-PANI/GO, b-PDPA/GO, and b-PT/DPA/GO increases to 0.85 nm, 0.80 nm, and 0.81 nm, respectively (Figure 2c). It should be noted that the content of the three intercalated polymers is exactly the same. We believe that b-PANI/GO and b-PT/DPA/GO exhibiting slightly larger interlayer spacings is due to the fact that their agglomerates create greater steric hindrance, causing the GO nanosheets to be spread apart.

Moreover, the thermal stability of the above composite precursors was tested. As shown in Figure 2d, the thermal decomposition of GO mainly occurs in two distinct stages. The first stage takes place around 30 °C~120 °C and primarily involves the slight thermal reduction of GO and the elimination of a few weak oxygen-containing groups [38], and the weight loss rate of GO in this stage is 17.04%. The second stage occurs at 120 °C to 1000 °C and primarily involves the further decomposition of abundant groups in GO [38,39], and the final residual carbon rate of GO is 27.65%. In comparison, the weight loss rates in the first stage for b-PANI/GO, b-PDPA/GO, and b-PT/DPA/GO are 15.37%, 14.37%, and 15.98%, and their residual carbon rates are 27.87%, 36.76%, and 29.79%, respectively. From these results, it can be seen that b-PDPA/GO shows superior thermal stability with an increased residual carbon rate of 9.11% compared to pure GO. This result is inconsistent with the thermal stability of pure polymers in Figure 1e. We believe this is primarily related to the compact stack structure of b-PDPA/GO, and the corresponding mechanism will be discussed in the subsequent section.

**Figure 3:**
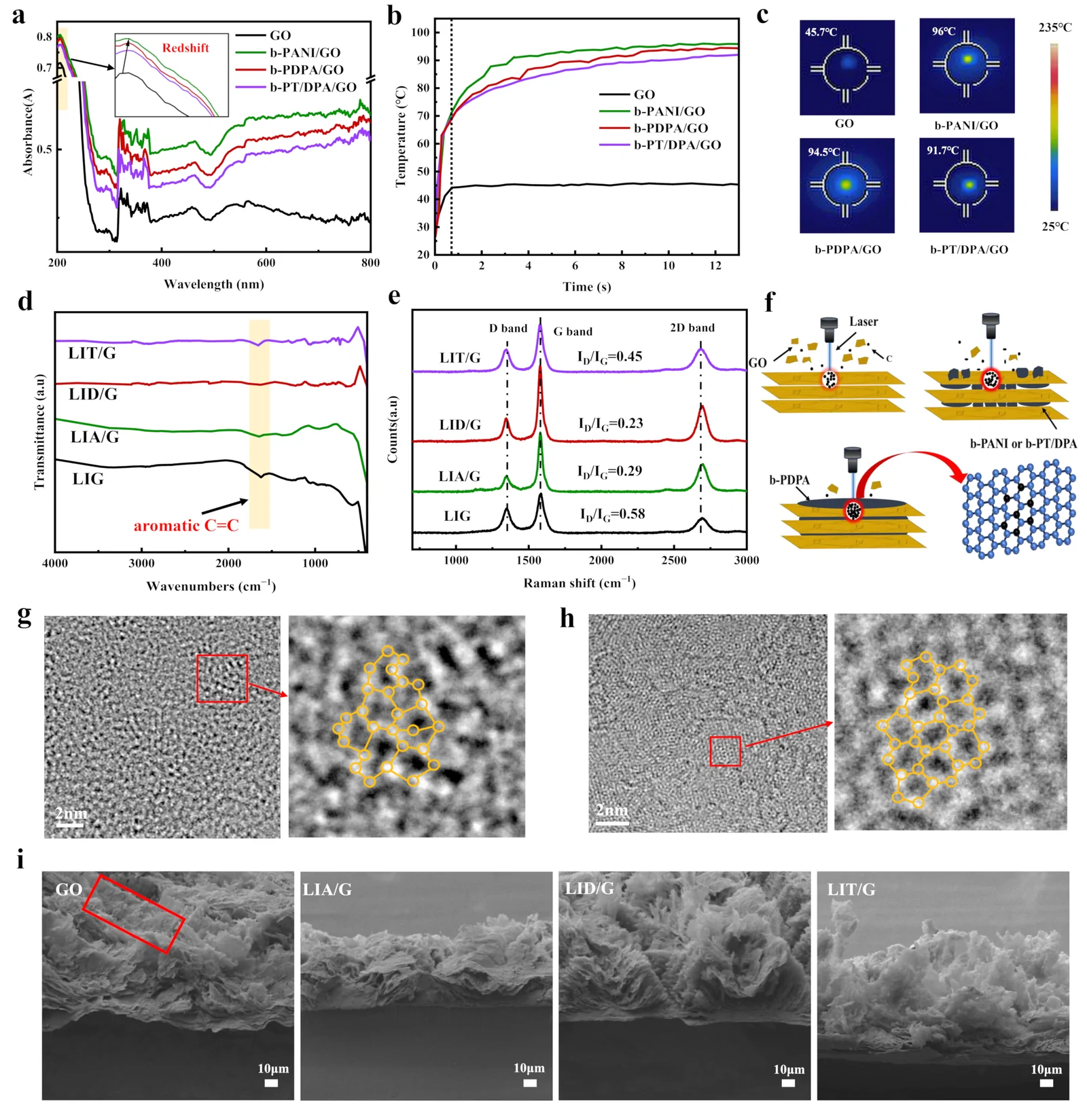
(**a**) The UV-Vis spectra of different composite precursors. (**b**) The temperature rise curves of different composite precursors under same laser irradiation. (**c**) The maximum temperature of different composite precursors under same laser irradiation. (**d**) The FTIR curves of different LIGs. (**e**) The Raman curves of different LIGs, and the corresponding I_D_/I_G_ values are average values from three parallel samples. (**f**) The mechanism schematic diagram for preparing different LIGs. (**g**,**h**) The HRTEM of LIG and LID/G. (**i**) The cross-section SEM of different LIGs.

To enhance the restoration effect for LIG, the superior photothermal capacity of precursors is essential. By utilizing the strong light absorption of P-PAs, this study aims to improve the photothermal-conversion performance of GO-based precursors. We characterized the light absorption of the above composite precursors. In Figure 3a, the results show that pure GO displays a weak light-absorbing capacity, and the corresponding aniline-based monomer-intercalated GO also shows no improved optical absorbance (Figure S4). In comparison, the absorption capability of b-PANI/GO, b-PDPA/GO, and b-PT/DPA/GO is significantly strengthened. Specifically, their absorbances are increased by 10.39%, 7.12%, and 4.95% at 450 nm wavelength, respectively, compared to that of GO. This is consistent with the changes in color in specimens (the inset in Figure 2a). Besides, with the incorporation of P-PAs, the characteristic absorption peak of GO exhibits an obvious redshift from 203.12 nm to 206.10 nm, which is predominantly attributed to the conjugated system of GO and P-PAs [38]. Based on the above property, under the same laser at 450 nm, the heating rates of b-PANI/GO, b-PDPA/GO, and b-PT/DPA/GO are 2.8, 2.3, and 2.2 times higher than that of pure GO, respectively, which indicates that the P-PAs make GO precursors respond more quickly to light, as shown in Figure 3b. Furthermore, the maximum temperatures of the GO, b-PANI/GO, b-PDPA/GO, and b-PT/DPA/GO were 45.7 °C, 96.0 °C, 94.5 °C, and 91.7 °C, respectively, which indicates that the P-PAs can trigger a higher laser-induced temperature (Figure 3c).

By means of the enhanced photothermal effect, the above composite precursor was adopted to prepare high-quality LIG via an available visible laser (wavelength ~450 nm, power ~100 mW, scanning speed ~657 mm s^−1^, spot size ~50 μm, line spacing ~0.01 mm, energy density ~0.015 J/mm^2^, and number of passes ~1). As a result, the graphene obtained from laser-induced GO, b-PANI/GO, b-PDPA/GO, and b-PT/DPA/GO are named LIG, LIA/G, LID/G, and LIT/G, respectively. The FTIR spectra of all graphene samples show hardly any sharp characteristic peaks across the tested wavenumber range, indicating that various oxygen- and nitrogen-containing functional groups have been efficiently removed through laser treatment (Figure 3d). Moreover, LIG, LIA/G and LIT/G exhibit a faint peak attributable to aromatic C=C stretching vibrations around 1630 cm^−1^, but LID/G shows no discernible peak at this position. We suppose this demonstrates a large-scale improved aromatic structure in LID/G, which endows a favorable molecular symmetry and thereby weakens the vibrational intensity of C=C bonds.

The above inference was confirmed by Raman testing, which further characterized the differences in defect levels of the obtained graphene. As shown in Figure 3e, it is clearly observed that all samples exhibit prominent D, G, and 2D peaks in their Raman spectra, which are the typical peaks of graphene nanosheets. Specifically, the D peak appears at approximately 1346 cm^−1^, arising from lattice defects and disorder. The G peak, located near 1580 cm^−1^, is generated by the in-plane C–C translational vibration of sp^2^-hybridized carbon atoms, indicating the symmetry and structural order of the GO precursor. Therefore, the I_D_/I_G_ ratio was used to quantitatively evaluate the defect level. The results revealed that I_D_/I_G_ is markedly reduced owing to the presence of the intercalated P-PAs. In particular, LID/G exhibited the lowest I_D_/I_G_ value of 0.23, compared with LIA/G (I_D_/I_G_ ~0.29), LIT/G (I_D_/I_G_ ~0.45), and LIG (I_D_/I_G_ ~0.58), indicating the lowest I_D_/I_G_ with a 60% reduction compared with LIG. Besides, the crystalline size of LID/G was calculated to be 83.59 nm (Table S1). This well-defined graphene structure was also verified by high-resolution transmission electron microscopy (HRTEM). Compared with that in traditional LIG, the graphene sheets in LID/G display much more ordered crystalline frameworks and improved benzene ring architectures (Figure 3g,h). The improved quality observed in LID/G provides a potential structural explanation for its excellent conductivity.

To explain the above phenomena, a schematic diagram is illustrated in Figure 3f. For pure GO, because of the weak photothermal effect, the laser-induced temperature is too low to repair the defects in LIG. In the case of b-PANI/GO and b-PT/DPA/GO, despite the enhanced photothermal performance, their loose-stacked state permits the escape of laser-induced carbon radicals, which are considered the main reagents for repairing graphene defects [40,41], and without barrier from air, the carbon radicals will also be consumed by O_2_, which all suppress the restoration effect; for the b-PDPA/GO system, owing to the strong photothermal effect and compact structure, the carbon radicals will be abundantly generated and prevented from escaping, so that they are maximally utilized for defect repair, which leads to a more ordered structure in LID/G. The isolation effect of the dense stack was supported by X-ray photoelectron spectroscopy (XPS) tests, which revealed that the O/C ratio of LID/G (1.38%) was much lower than that of LIG (2.78%), LIA/G (1.9%) and LIT/G (2.29%) (Figure S6). The blocking effect of the intercalated b-PDPA for carbon radicals agrees with the highest residual carbon rate of b-PDPA/GO in Figure 2d. Besides, despite using the identical intercalated polymer, owing to the loose interlayer bonding (Figure S3), LIGs prepared from laser-induced APS-initiating PANI/GOs, APS-initiating PDPA/Gos, and APS-initiating PT/DPA/GOs show high I_D_/I_G_ values of 0.54, 0.32, and 0.42, respectively (Figure S7). This degradation of LIG quality stemming from the loose stacking further agrees with the as-proposed isolation effect.

The introduction of P-PAs also influences the microstructure of the as-prepared graphene. As shown in Figure 3i, serious etching occurs in LIG owing to its low absorbance, so that many grooves appear (red box). LID/G exhibits a fluffier 3D aerogel structure than that of LIA/G and LIT/G, in which graphene sheets are better exfoliated. We attribute this phenomenon to the more homogeneous composite of b-PDPA and GO, so the thermally decomposed polymer consequently props apart the stacked graphene sheets. This interconnected network of aerogel will facilitate good conductivity for the subsequently formed TG. Therefore, b-PDPA will serve as the optimal intercalator for GO, and the corresponding graphene aerogel is prepared in the following study.

### 2.3. Cooperative Effect for LIG via b-PDPA and Fe^2+^

**Figure 4:**
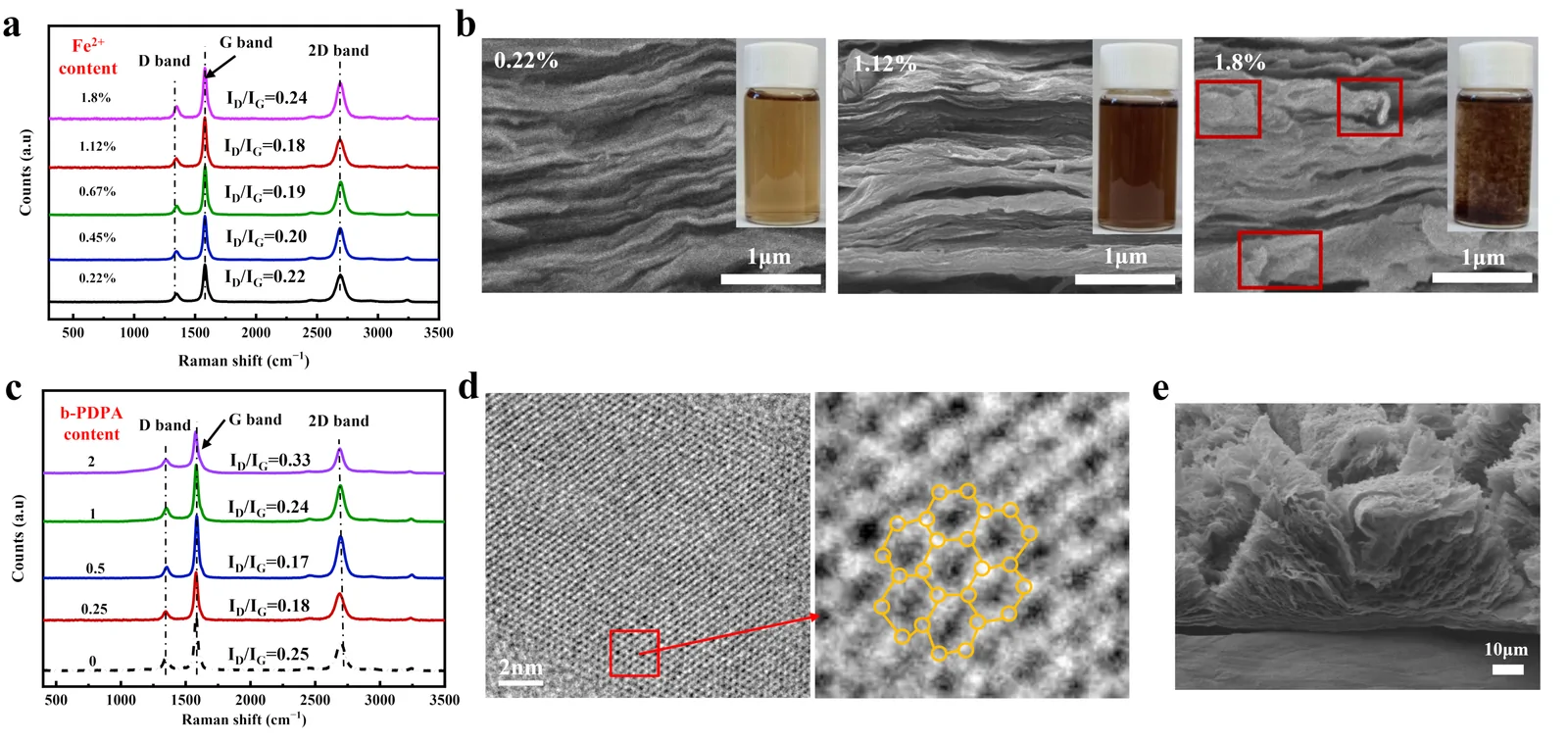
(**a**) The Raman curves of LID/G with different Fe^2+^ contents, and the corresponding I_D_/I_G_ values are average values from three parallel samples. (**b**) The cross-sectional SEM of b-PDPA/GO with different Fe^2+^ contents, and the insets show the corresponding states of the previous solutions. (**c**) The Raman curves of LID/G with different b-PDPA contents, and the corresponding I_D_/I_G_ values are average values from three parallel samples. (**d**) The HRTEM of LID/G with Fe^2+^ content of 1.12% and b-PDPA content of 0.5. (**e**) The cross-sectional SEM of LID/G with Fe^2+^ content of 1.12% and b-PDPA content of 0.5.

Many investigations reveal that appropriate metal ions have a promotional effect on repairing LIG [4,15,16,17,18,19,42]. Herein, common Fe^2+^ is incorporated as a catalyst to further improve the quality of the as-prepared graphene. The effect of Fe^2+^ content on the conversion of b-PDPA/GO into LID/G was first studied. Various contents of Fe^2+^, including 0.22%, 0.45%, 0.67%, 1.12%, and 1.80%, corresponding to the weight ratio of Fe^2+^ relative to the composite precursors, were introduced into b-PDPA/GO. The results reveal that after identical laser treatment, the I_D_/I_G_ of the obtained LID/G first gradually decreases as the Fe^2+^ increasing, reaches a minimum of 0.18 for 1.12% content, and then increases to 0.24 for 1.8% content. This indicates that when the Fe^2+^ content is 1.12%, the obtained LID/G exhibits the optimal defect-repairing effect, which has a L_a_ of 106.80 nm (Table S2). The enhanced repair effect of LIG at low Fe^2+^ loadings is mainly attributed to the catalytic carbonization, which has been verified in reported studies [43]. In contrast, the decreased repair capability at high ion loadings originates from excessive Fe^2+^ causing the flocculation and precipitation of b-PDPA and GO nanosheets, as shown in the inset of Figure 4b. This reduces the compactness of the stacked layer structure, and many large aggregates occur (red boxes in Figure 4b). Based on the previously stated isolation effect, this loose state will result in poor blocking and a low repair degree for LID/G. According to the above experimental results, 1.12% Fe^2+^ was utilized in the subsequent study.

Next, the composite precursors containing 1.12% Fe^2+^ with different weight ratios of b-PDPA to GO of 0.25, 0.5, 1, and 2 were fabricated, and the defect degree of the corresponding LID/Gs obtained through laser treatment was characterized. In Figure 4c, the results reveal that the LID/G fabricated from the b-PDPA ratio of 0.5 possesses the best repair degree, with an I_D_/I_G_ reaching 0.17. Its crystalline size reaches 113.09 nm (Table S2). Such a low defect level has barely been achieved in the reported LIG materials yet [18,19,22,44,45]. Besides, the FWHM of the 2D peak is 97.68 cm^−1^, which is very close to the Lorentzian FWHM of five-layer graphene, which indicates a thin graphene layer structure [46,47]. This demonstrates that fully separated graphene nanosheets are achieved, which agrees with its enhanced fluffy porous structure in Figure 4e. HRTEM further confirms its well-defined crystalline lattice. As presented in Figure 4d, extensive regular lattice structures together with plentiful six-membered rings are clearly observed, closely resembling the ideal graphene framework. In addition, it should be noted that Fe^2+^ and b-PDPA exhibit a prominent cooperative effect for improving LID/G. In the absence of b-PDPA, the I_D_/I_G_ of LIG via Fe^2+^-catalyzed pure GO is as high as 0.25 (Figure 4c). This demonstrates that the isolating effect of b-PDPA can also deliver a promotion in ion-catalyzed LIG systems. It should be noted that a small amount of Fe is present in the as-obtained LID/Gs (Figure S10).

### 2.4. Thermal-Conductive LID/G Gel and Its Efficiency in Interfacial Heat Transfer

**Figure 5:**
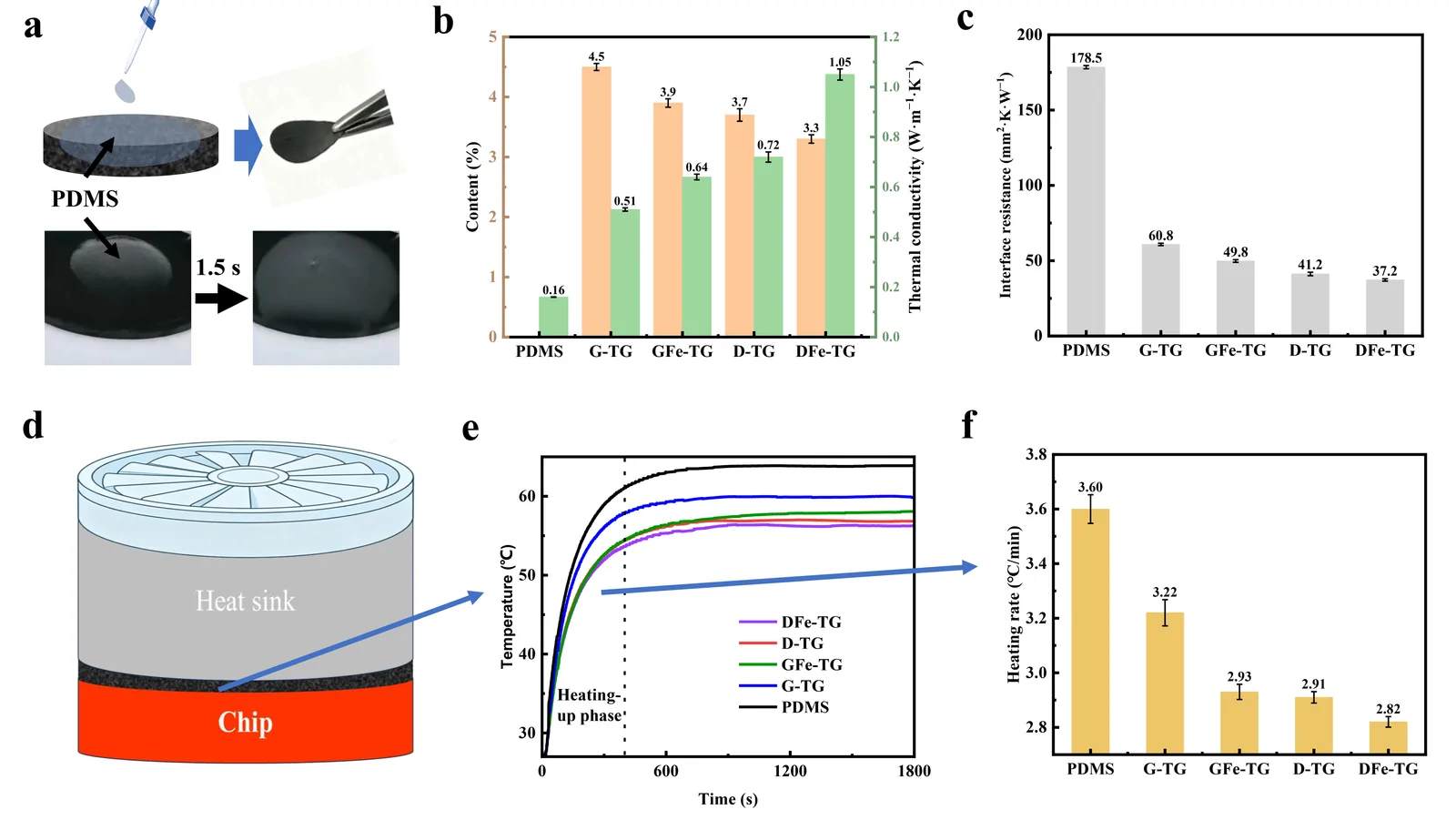
(**a**) Schematic diagram and photographs of PDMS resin infiltration into graphene aerogel. (**b**) The thermal conductivity and graphene content of different TGs, and the corresponding values are average values from three parallel samples. (**c**) The interface thermal resistance of different TGs, and the corresponding values are average values from three parallel samples. (**d**) Heat transfer schematic diagram for heated chip. (**e**) The heating-up curves of chip with different TGs involved. (**f**) The heating rate of the chip with different TGs involved, and the corresponding values are average values from three parallel samples.

Owing to the improved graphene quality, the as-prepared LID/G is theoretically endowed with enhanced heat-conducting performance. For comparison, four groups of graphene aerogels were composited with PDMS: LIG, LIG with only Fe^2+^ content of 1.12%, LID/G with only b-PDPA content of 0.25, and LID/G with both Fe^2+^ content of 1.12% and b-PDPA content of 0.5 (possessing the optimum defect-restoration condition in this study). Their corresponding I_D_/I_G_ ratios are 0.58, 0.25, 0.23, and 0.17, which clearly demonstrate the cooperative effect between b-PDPA and Fe^2+^. The obtained corresponding thermal-conductive gels were named G-TG, GFe-TG, D-TG, and DFe-TG. Benefiting from graphene’s porous architecture and hydrophobic-oleophilic characteristics, PDMS resin can rapidly infiltrate into the interior of aerogels in the fabrication process, which enables homogeneous TGs (Figure 5a). The graphene content in all TGs was determined from the mass difference before and after compositing with PDMS. The results reveal that DFe-TG possesses the lowest graphene content of 3.3 wt%, which is attributed to its fluffy microstructure and well-exfoliated graphene nanosheets, as stated previously. Moreover, benefiting from high-quality graphene and an interconnected conductive network, DFe-TG shows the highest thermal conductivity of 1.05 W·m^−1^·K^−1^, which is 6.5-fold, 2.1-fold, 1.6-fold, and 1.5-fold larger than that of pure PDMS, G-TG, GFe-TG, and D-TG, respectively (Figure 5b). Attaining such high thermal conductivity under this low graphene content remains challenging for traditional LIG-based gels [48,49,50,51,52]. In summary, the defect-repaired LID/G aerogel by b-PDPA and Fe^2+^ in this work exerts a prominent effect on improving the thermal conductivity of polymer gels.

Considering the high thermal conductivity and flexibility, DFe-TG possesses a natural advantage for interfacial heat transfer. The interfacial thermal resistance of the above gels was measured using an interfacial heat transfer measurement system under ambient conditions with an applied pressure of 0.5 MPa via adjusting the bolt torque. The relevant characterization procedure was documented in our earlier publication [23]. The corresponding interfacial thermal resistance values are average values from three parallel samples, and their error bars represent the standard deviation. The results demonstrate that the interfacial thermal resistance of pure PDMS with a thickness of 30 μm and diameter of 30 mm reaches 178.5 mm^2^·K·W^−1^, which originates from its thermal-insulating property. Once composited with graphene aerogel, the gels show an obvious decline in interfacial thermal resistance. The corresponding values for G-TG, GFe-TG, D-TG and DFe-TG are 60.8 mm^2^·K·W^−1^, 49.8 mm^2^·K·W^−1^, 41.2 mm^2^·K·W^−1^ and 37.2 mm^2^·K·W^−1^ (Figure 5c). In other words, the interfacial thermal resistance of DFe-TG is only 21% of pure PDMS and 61% of the conventional LIG-based gels. This lays the foundation for achieving efficient interfacial heat-transfer performance via DFe-TG. The heat-transfer ability of DFe-TG on a heat chip (heating power of 3 W) was explored using a heat dissipation device under ambient conditions, as illustrated in Figure 5d. The diameters of the sample and chip were 30 mm, and a miniature thermocouple was attached to the chip surface. The corresponding heating-up rates were average values from three parallel samples, and their error bars represented the standard deviation. The results demonstrate that under identical conditions, the heating-up rate of the chip with DFe-TG decreases by 21.7% and 12.4%, and its final steady-state temperature drops by 7.73 °C and 6.82 °C compared that with pure PDMS and G-TG involved. Moreover, the thermal conductivity, interfacial thermal resistance, and tensile strength of DFe-TG show no obvious degradation after 120 h of service for chip heat dissipation (at about 55 °C), which demonstrates good long-term durability (Figure S11). It proves that DFe-TG possesses prominent advantages for thermal dissipation in a high-heat-flux environment such as chips, batteries, and laser devices.

## 3. Conclusions

In conclusion, by modifying the molecular structure and reducing the molecular weight, the solubility of poly-phenylamines was improved significantly. Accordingly, composite precursors containing GO nanosheets and these polymers were prepared via evaporation-induced co-assembly. By means of the intercalation of this polymer, the photothermal effect and interlayer bonding in composite precursors were enhanced. Based on this, the I_D_/I_G_ of the obtained LIG was reduced by 60%, and a mechanism of isolation effect by the compact intercalation was proposed in this study. Moreover, a cooperative effect with Fe^2+^ was introduced to further improve graphene quality in LIG. Finally, a high-quality graphene aerogel of LID/G with the lowest I_D_/I_G_ ratio of 0.17 was obtained. By means of the repaired graphene nanosheets and the fluffy network, the thermal-conductive gels composed of PDMS and LID/G reached a high thermal conductivity of 1.05 W·m^−1^·K^−1^ and a low interfacial thermal resistance of 37.2 mm^2^·K·W^−1^ at the graphene content of only 3.3 wt%. This facile precursor-preparing method and accessible laser treatment greatly facilitate scalable fabrication. Moreover, it partially overcomes the limitations of conventional graphene defect repair that rely on extremely high-temperature treatment. Based on the above conclusions, we provide a new strategy and correlated theories for improving the quality of LIG, which are available for fabricating high-performance interfacial thermal-transport materials.

## 4. Materials and Methods

### 4.1. Materials

Aniline (99.5%), DPA (99%), TPA (98%), FeCl_2_·4H_2_O (98%), and n-Hexane (98%) were purchased from Shanghai Macklin Biochemical Technology Co. Ltd. (Shanghai, China). Pristine graphite powder (325 mesh), BPO (AR), and APS (AR, 98.5%) were purchased from Shanghai Aladdin Biochemistry Technology Co. Ltd. (Shanghai, China). Ethanol (AR) was obtained from Sinopharm Chemical Reagent Co. Ltd. (Shanghai, China). Deionized water was supplied by Pineak Instrumentation Equipment Co. Ltd. (Wuhan, China). SYLGARD^TM^ 184 Silicone Elastomer Base and SYLGARD^TM^ 184 Silicone Elastomer Curing Agent were purchased from DOW SILICONES DEUTSCHLAND GMBH (Wiesbaden, Germany). None of the chemicals were further purified.

### 4.2. Methods

#### 4.2.1. The Synthesis of P-PAs

500 mg of aniline-based monomers (aniline, DPA, or DPA: TPA = 100:4 by weight) were added to 12.5 mL of ethanol in an 80 °C water bath and stirred until the monomers were completely dissolved. Then dilute hydrochloric acid was added to adjust the pH to approximately 5. Next, 1 g of a different initiator, BPO or APS, was introduced, and the monomers were cured at 60 °C for 2 h. Finally, 37.5 mL of water was added to precipitate the product, and the P-PAs were obtained via high-speed centrifugation.

#### 4.2.2. The Preparation of P-PA/GO Composite Precursors

The GO aqueous dispersion was prepared from pristine graphite powder according to a modified Hummers’ method as reported in our previous paper [53,54]. A certain amount of ethanol was added to adjust the ethanol/water volume ratio at 9:1. The polymers (a-PANI, a-PDPA, a-PT/DPA, b-PANI, b-PDPA, or b-PT/DPA) or aniline-based monomers (aniline, DPA or DPA:TPA = 100:4 by weight), taking 25% of the GO concentration (3 mg mL^−1^, 15 mL), were added and heated at 80 °C for 2 h. The corresponding composite precursors were prepared by evaporating mixtures at 60 °C to form composite precursors with a diameter of 30 mm and thickness of about 1.8 μm.

#### 4.2.3. The Preparation of b-PDPA/GO Composite Precursors with Fe^2+^

**Figure 6:**
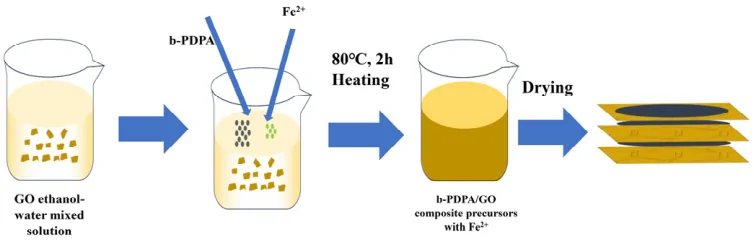
The schematic mechanism of GO/P-PA assembly.

A specific amount of FeCl_2_·4H_2_O (Various contents of 0.8%, 1.6%, 2.4%, 4.0%, and 6.4%, corresponding to the weight ratio of FeCl_2_·4H_2_O relative to the composite precursors) was introduced into the b-PDPA-GO mixture and stirred at 80 °C for 2 h. Then, the composite precursors were prepared by evaporating the mixture at 60 °C to form composite precursors with a diameter of 30 mm and a thickness of about 1.9 μm (Figure 6).

#### 4.2.4. The Preparation of LIGs and TGs

Laser carbonization was performed using a laser (wavelength ~450 nm, power ~100 mW, scanning speed ~657 mm s^−1^, spot size ~50 μm, line spacing ~0.01 mm, energy density ~0.015 J/mm^2^, and number of passes ~1) via direct laser engraving of composite precursors under ambient atmosphere. The obtained LIGs were fully impregnated with PDMS (SYLGARDTM 184 Silicone Elastomer Base: Curing Agent = 10:1) resin and cured at 60 °C for 4 h to yield TGs in a forced-air drying oven.

### 4.3. Characterization

The morphology of dried P-PAs was characterized using a M3230Bd series metallurgical microscope (Phenix, Shangrao, China). The morphology of the composite precursors and LIGs was characterized using a JSM-7500F scanning electron microscope (JEOL, Tokyo, Japan). The FTIR spectra of samples were characterized using a Nicolet 6700 series Fourier transform infrared spectrometer (TMO, Waltham, MA, USA). The Raman spectra of LIGs were characterized using a LabRAM Odyssey series Laser confocal Microscope Raman spectrometer with an excitation wavelength of 532 nm (TMO, Waltham, MA, USA). The corresponding I_D_/I_G_ values are average values from three parallel samples, and their error bars represent the standard deviation. We evaluated the *L_a_* of the obtained LIGs [21,55].

$$L_{a}=(2.4\times 10^{-10})\times \lambda_{ı}^{4}\times \frac{I_{G}}{I_{D}}$$

where λ_l_ (~532 nm) is the wavelength of the Raman laser. The interlayer spacing was characterized using XRD analysis with Cu Kα radiation (λ = 0.15418 nm) in the scan range of 2θ = 5–80° at a scan rate of 1°/min. The lattice structure of LIGs was characterized using HRTEM (JEM-F200, JEOL, Tokyo, Japan). The absorbance of samples was characterized using UV spectroscopy (Lambda 750 S, San Francisco, CA, USA). The molecular weight distribution of P-PAs was characterized using GPC (PL-GPC50, Agilent Technologies, Inc., Santa Clara, CA, USA). The surface temperature of the composite precursor was characterized using infrared thermography T420 (FLIR, Wilsonville, OR, USA). The thermal conductivity of the sample along the thickness direction was determined using a TCT716 series Heat Flux Meter (NETZSCH, Selb, Germany), and the diameter and thickness of the sample is 50 ± 1 mm and 0.03 mm, respectively. The corresponding thermal conductivity values are average values from three parallel samples, and their error bars represent the standard deviation. The tensile strength of TGs was evaluated using a universal testing machine at a strain rate of 200 mm min^−1^ (RGM-3005, Reger, Shenzhen, China), and the corresponding values are average values from three parallel samples, and their error bars represent the standard deviation.

## Data Availability

The data presented in this study are openly available in the article.

## Supplementary Materials

The following supporting information can be downloaded at https://www.mdpi.com/article/10.3390/gels12090835/s1. Figure S1: The FTIR curves of P-PAs. Figure S2: Molecular weight distribution diagrams of a-PDPA and a-PT/DPA. Figure S3: The SEM of the a-P-PA/GO composite precursors. Figure S4: The UV-Vis spectra of the monomer-intercalated GO composite precursors. Table S1: The L_a_s of a-LIGs. Figure S5: The error bar for I_D_/I_G_ of LIGs. Figure S6: The XPS of LIGs. Figure S7: The Raman curves of a-LIGs. Figure S8: The error bar for I_D_/I_G_ of LIGs with different Fe^2+^ content. Figure S9: The error bar for I_D_/I_G_ of LIGs with different b-PDPA content. Table S2: The L_a_s of LIGs. Figure S10: The XPS of LID/G with Fe. Figure S11: The tensile strength, thermal conductivity, and interfacial thermal resistance of DFe-TG after being used at about 55 °C for 120 h. Table S3: The quantitative comparison between this TG and reported thermal-conductive composites.

## Abbreviations

The following abbreviations are used in this manuscript:

P-PAs	Poly-Phenylamines
GO	Graphene oxide
LIG	Laser-induced graphene
a-PANI	APS initiating PANI
b-PANI	BPO initiating PANI
b-PDPA	BPO initiating PDPA
b-PT/DPA	BPO initiating copolymerization containing DPA and TPA (100:4 by weight)
LIA/G	Laser-induced b-PANI/GO
LID/G	Laser-induced b-PDPA/GO
LIT/G	Laser-induced b-PT/DPA/GO
TG	Thermal-conductive gel
G-TG	Composite LIG with PDMS to fabricate corresponding thermal-conductive gel
GFe-TG	Composite LIG with only Fe^2+^ contents of 1.12% with PDMS to fabricate corresponding thermal-conductive gel
D-TG	Composite LID/G with only b-PDPA contents of 0.25 with PDMS to fabricate corresponding thermal-conductive gel
DFe-TG	Composite LID/G with both Fe^2+^ contents of 1.12% and b-PDPA contents of 0.5 with PDMS to fabricate corresponding thermal-conductive gel
