# Supplementary material for: Cooperative Repair for Laser-Induced Graphene via Modified Poly-phenylamine and Fe2+ for Thermal-Conductive Gels
Source: Gels. 2026 Sep 11;12(9):835. doi: 10.3390/gels12090835 (PMC13606344; doi:10.3390/gels12090835)
Supplement: Supplementary file 1 [file gels-12-00835-s001.zip › gels-4525693-supplementary.pdf]

Supporting Information

Cooperative repair for Laser-induced Graphene via Modified Poly-phenylamine and Fe^{2+} for Thermal-Conductive Gels

AUTHOR NAMES: Nan Jiang, Guomin Ding*, Bowen Yang, Shuai Liu, Luyao Wang, Zihan Li, Xu Han, Qilin Mei

AUTHOR ADDRESS: School of Materials Science and Engineering, Wuhan University of Technology, 122 Luoshi Road, Wuhan 430070, China

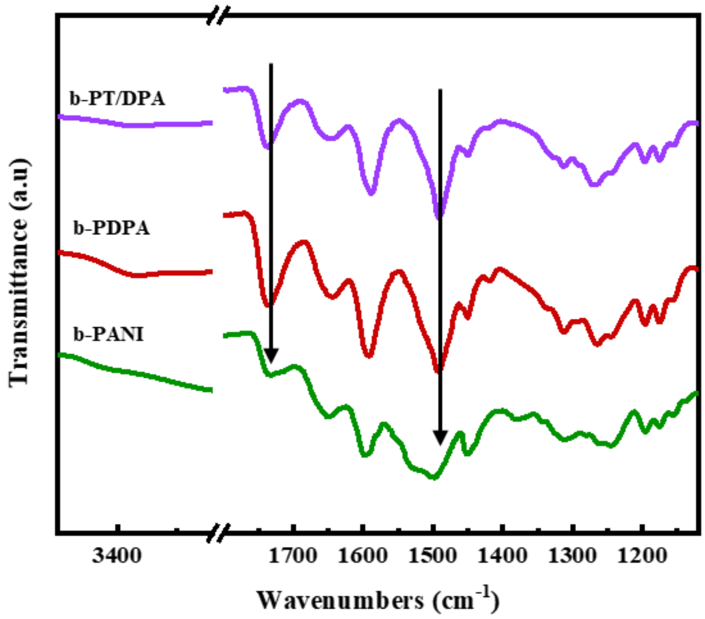

Figure S1. The FTIR curves of P-Pas.

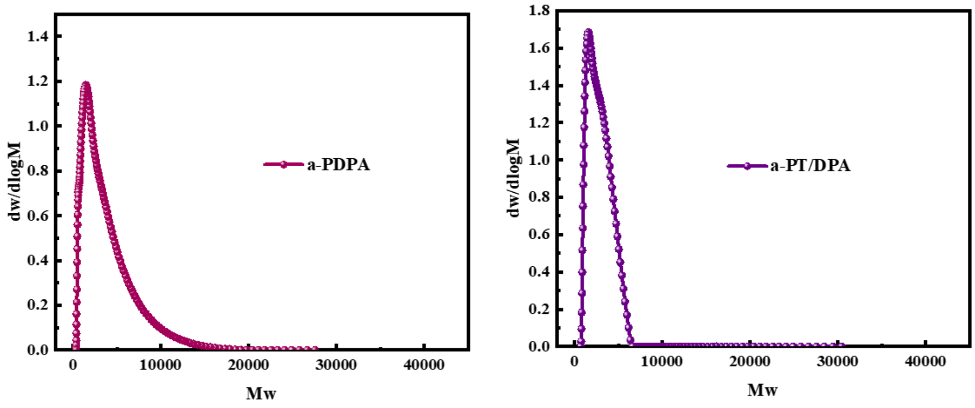

Figure S2. Molecular weight distribution diagrams of a-PDPA and a-PT/DPA.

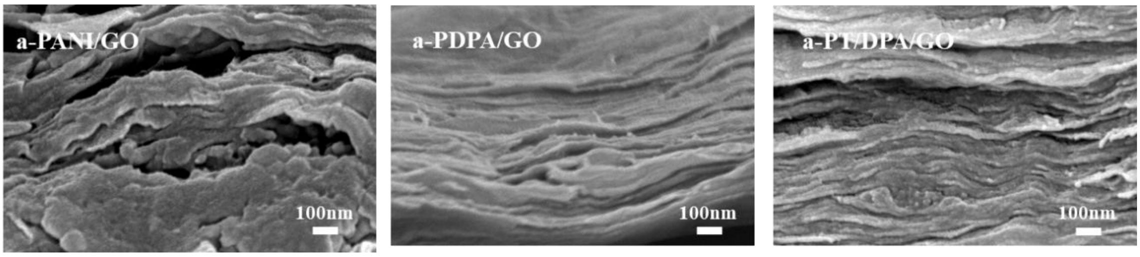

Figure S3. The SEM of the a-P-PA/GO composite precursors.

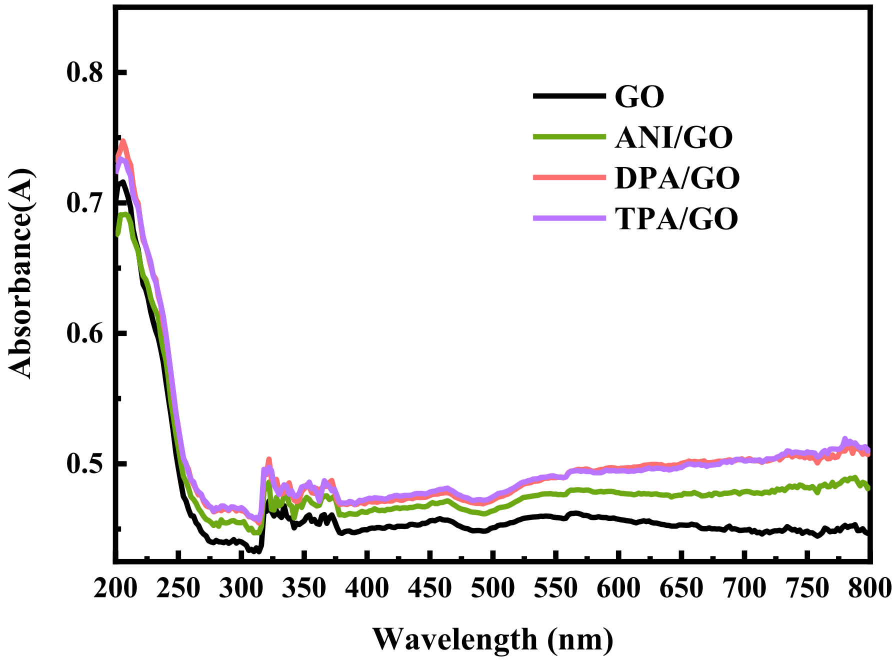

Figure S4. The UV-Vis spectra of the monomer-intercalated GO composite precursors.

Table S1. The L_as of a-LIGs

LIGs	L_a (nm)
LIG	33.15
LIA/G	66.29
LID/G	83.59
LIT/G	42.72

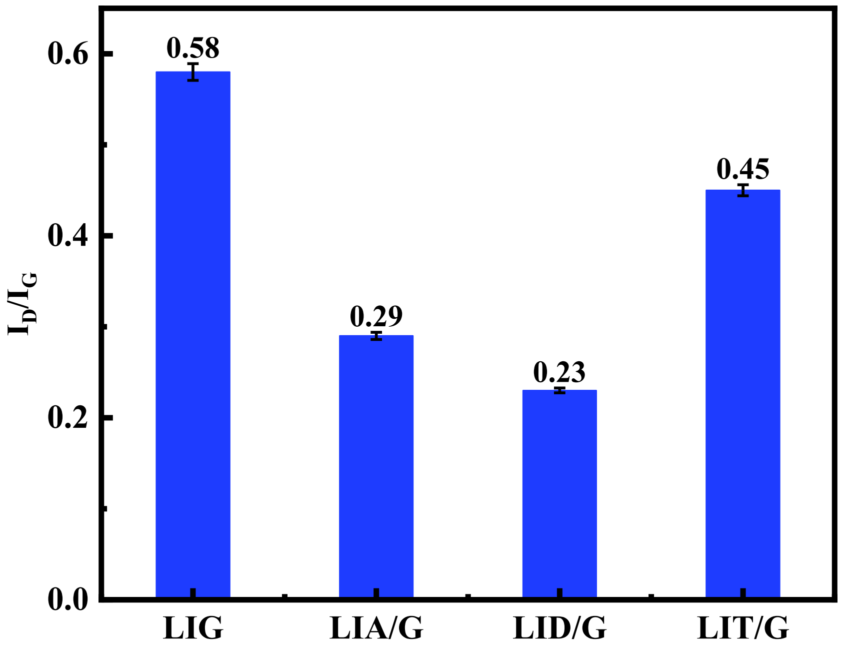

Figure S5. The error bar for I_D/I_G of LIGs

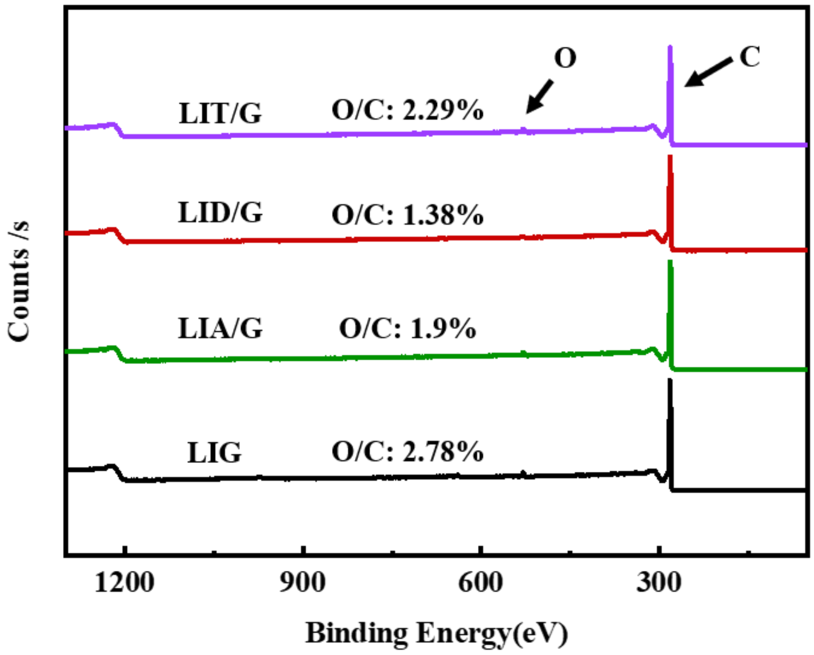

Figure S6. The XPS of LIGs

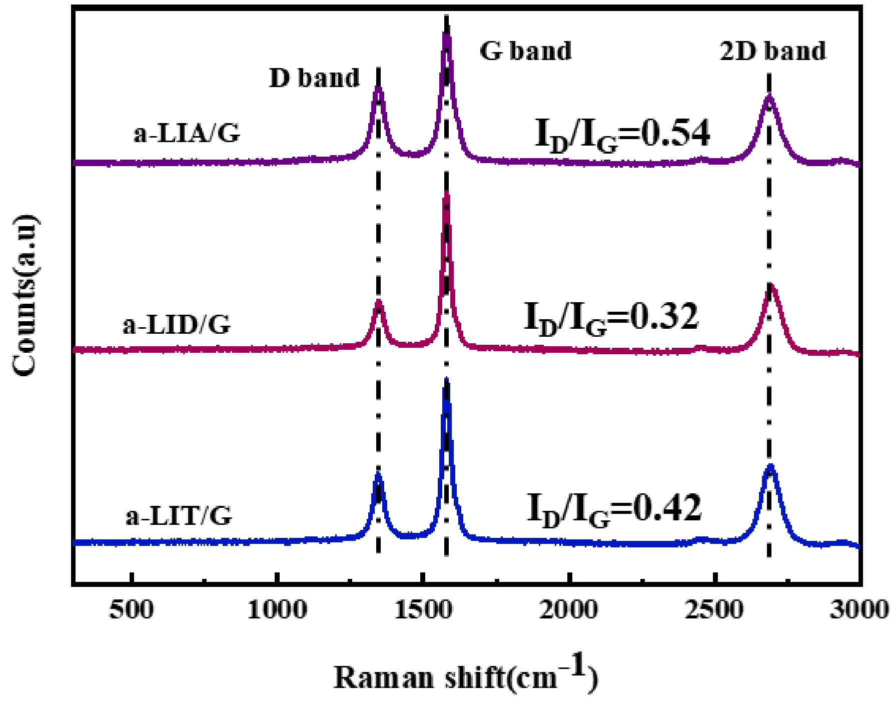

Figure S7. The Raman curves of a-LIGs

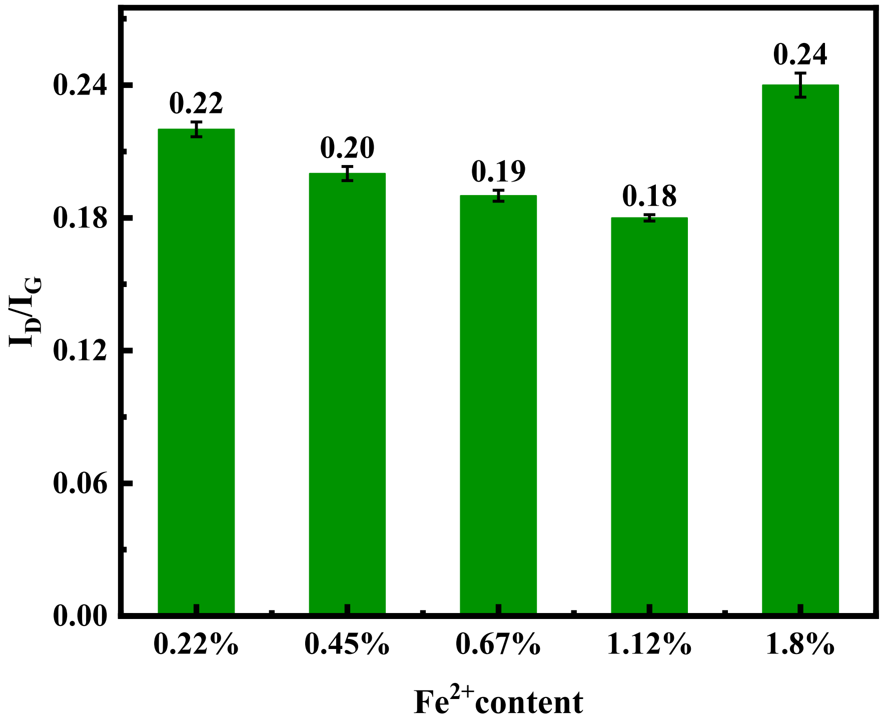

Figure S8. The error bar for I_D/I_G of LIGs with different Fe^{2+} content

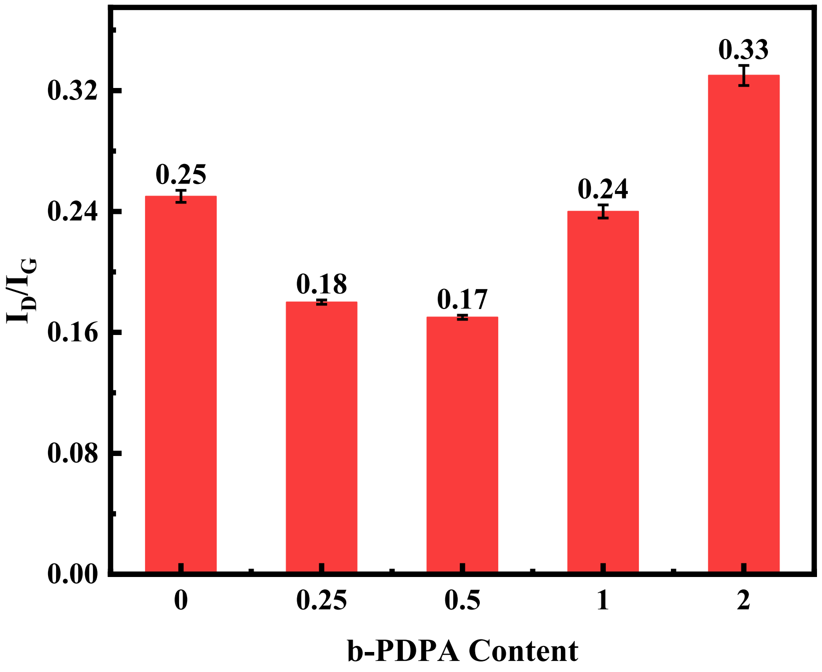

Figure S9. The error bar for I_D/I_G of LIGs with different b-PDPA content

Table S2. The L_as of LIGs

Fe^{2+} content (%)	L_a (nm)	b-PDPA content (%)	L_a (nm)
0.22	87.38	0	76.90
0.45	96.12	0.25	106.80
0.67	101.18	0.5	113.09
1.12	106.80	1	80.10
1.8	80.10	2	58.26

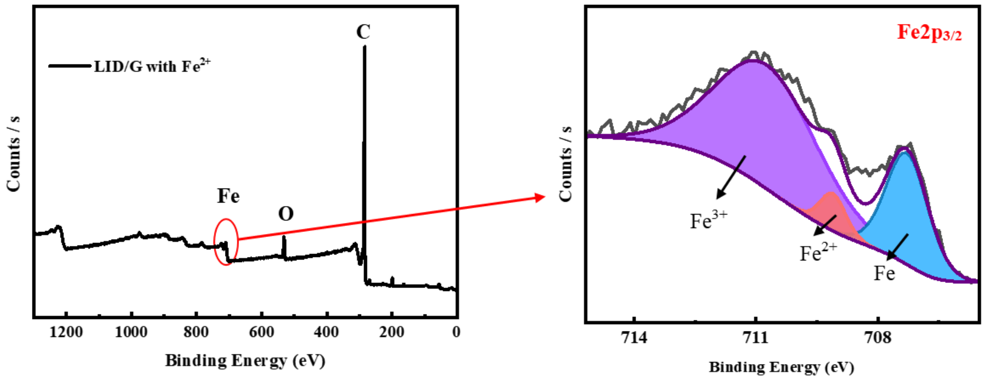

Figure S10. The XPS of LID/G with Fe

The residual Fe contents in LID/G is about 1.3 %, which contains metallic Fe, Fe^{2+}, and Fe^{3+}. The increased relative Fe content is mainly attributed to the reduced mass of the precursor after laser-induced graphene conversion. The different Fe states are mainly because the carbon formed by laser irradiation partially reduces Fe^{2+} under the high laser temperature. Meanwhile, the residual Fe^{2+} is gradually oxidized to Fe^{3+} in air. The corresponding contents of the above three species were calculated by deconvoluting the Fe XPS curves: 25.5 % of Fe, 5.5 % of Fe^{2+}, and 69.0 % of Fe^{3+}.

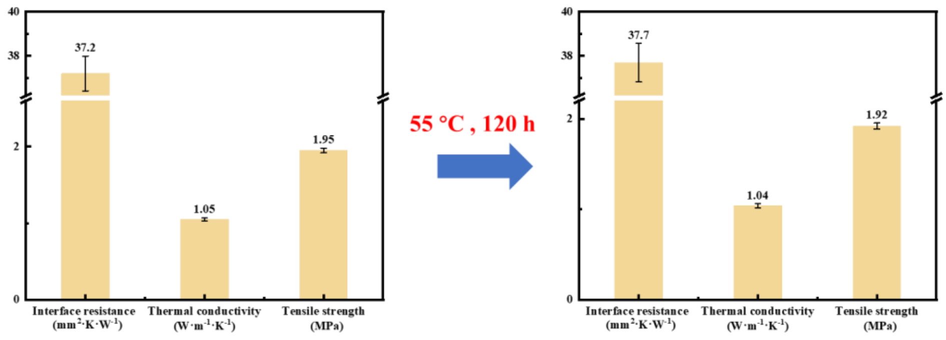

Figure S11. Tensile strength, thermal conductivity, and interfacial thermal resistance of DFe-TG after use at about 55 °C for 120 h.

Table S3. The quantitative comparison between this TG and reported thermal-conductive composites.

Matrix Material	Filler Material	Filler Loading	Thermal Conductivity (W·m^{-1}·K^{-1})	Ref
PDMS (as supporting matrix)	Graphene (GPE)	0.5 wt%	0.3065	48
PDMS	3D-printed graphene skeleton (HT-rGO)	2.75wt%	0.83	49
PDMS	Graphene aerogel (GA)	1 wt%	0.58	50
PDMS	Vertically-oriented GNP-BN hybrid skeleton (GNP:BN = 1:3)	5.2 wt%	1.16	51
PDMS	Functionalized graphene (f-G)	2 wt%	0.761	52
PDMS	LID/G	3.3 wt%	1.05	This work
